# Postoperative Outcomes of Robotic Versus Conventional Surgery in a Multi-Cancer Cohort: A Retrospective Cohort Study

**DOI:** 10.3390/jcm15207846

**Published:** 2026-10-12

**Authors:** Jaehyeong Cho, Hyoshuk Kim, Chi-Heum Cho

**Affiliations:** 1 Department of Research, Keimyung University Dongsan Medical Center, 1035 Dalgubeol-daero, Dalseo-gu, Daegu 42601, Republic of Korea; 2 Department of Digital Health, Samsung Advanced Institute for Health Sciences and Technology (SAIHST), Sungkyunkwan University, 115 Irwon-ro, Gangnam-gu, Seoul 06355, Republic of Korea; 3 Department of Obstetrics and Gynecology, Keimyung University School of Medicine, 1095 Dalgubeol-daero, Dalseo-gu, Daegu 42601, Republic of Korea

**Keywords:** robotic surgical procedures, oncologic surgery, length of stay, comparative effectiveness research, minimally invasive surgical procedures, patient readmission, health resources

## Abstract

**Background/Objectives**: Evidence on robotic surgery is largely procedure-specific and depends on the comparator. We examined associations with postoperative outcomes, prioritizing colorectal and uterine cancer and distinguishing laparoscopic from open comparators. **Methods**: This retrospective cohort included 2139 patients undergoing elective surgery for colorectal, uterine, prostate, or renal cancer at a tertiary referral center in 2019–2023. The primary analysis included 1232 colorectal or uterine cancer patients undergoing robotic (*n* = 462) or laparoscopic (*n* = 770) surgery. Generalized linear models adjusted for age, sex, cancer type, and T category/FIGO stage group; operative time and staffing were added in sensitivity analysis. Absolute differences were estimated by G-computation. The four-cancer pooled analysis was secondary. **Results**: Robotic surgery was associated with a shorter hospital stay (length-of-stay ratio 0.78; 95% CI 0.73–0.83; adjusted difference 2.34 days), which persisted with operative-time/staffing and calendar-year adjustment. Rehospitalization within 30 days after surgery was higher overall (adjusted odds ratio [aOR] 1.82; 95% CI 1.34–2.47), but differed by cancer: aOR 2.50 (95% CI 1.77–3.53) for colorectal and 0.38 (95% CI 0.16–0.90) for uterine cancer (interaction *p* < 0.001). Estimates for 30-day mortality, postoperative complications, and surgical site infection were imprecise. **Conclusions**: Rehospitalization differed in direction between these cancers, indicating that cancer type and comparator selection materially affect inference. These associations do not establish a platform-specific benefit or transferable bed-day savings.

## 1. Introduction

Robotic surgery has been increasingly adopted across oncologic procedures [1,2], with systematic reviews assessing outcomes across several cancer types [3,4]. Technical advantages include enhanced three-dimensional visualization, improved instrument articulation, and tremor filtration [5]. In selected procedures, comparative studies have reported lower conversion rates and shorter postoperative stay [4,6,7]. Length of stay (LOS) is an important measure of hospital resource use, but differences in LOS should be considered alongside rehospitalization.

Evidence from individual procedures may not apply across cancer types. Comparisons with laparoscopic and open surgery also address different clinical questions, and pooling these approaches may obscure differences in outcomes. Comparative studies and pooled analyses have reported variable short-term outcomes, leaving uncertainty about hospital use when robotic surgery is compared with a specific conventional approach [8,9,10]. Interpretation of hospital resource use must also account for the acquisition and operating costs of robotic systems [11].

To address these gaps, we conducted a retrospective cohort study at a tertiary referral center. The primary analysis compared robotic and laparoscopic surgery for colorectal and uterine cancer, where both approaches were represented. We examined associations with perioperative outcomes and estimated absolute differences in LOS and 30-day rehospitalization to inform interpretation of hospital resource use. Comparisons with open surgery and pooled analyses of colorectal, uterine, prostate, and renal cancer were secondary. Long-term oncologic outcomes were not assessed.

## 2. Materials and Methods

### 2.1. Study Design and Data Source

This retrospective cohort study analyzed consecutive patients who underwent elective oncologic surgery at Keimyung University Dongsan Hospital, a tertiary referral center in Daegu, Republic of Korea. Electronic medical records were reviewed to identify all procedures performed for colorectal, uterine, prostate, or renal cancer between 2019 and 2023.

### 2.2. Patient Selection

Patients diagnosed on or after 1 January 2019 who underwent elective oncologic surgery for one of these four malignancies were considered for inclusion. Although records extended from January 2018, the index date was set to January 2019 to ensure at least 12 months of prior administrative history. Patients were excluded for concurrent diagnosis of more than one index cancer, coexistence of robotic and laparoscopic procedure codes within the same operative record, duplicate administrative identifiers, missing diagnosis code after linkage, unverifiable disease stage, or irreconcilable inconsistency between admission, operative, and discharge records identified during data quality review (*n* = 2). After sequential exclusions, 2139 patients were retained (Supplementary Methods). The primary analysis included 1232 patients with colorectal or uterine cancer undergoing robotic or laparoscopic surgery; 70 open procedures in these cancers were included only in the secondary open comparison.

### 2.3. Surgical Approach Classification

Surgical procedures were classified according to the operative platform used. The robotic group comprised all procedures performed with the da Vinci robotic surgical system (Intuitive Surgical, Sunnyvale, CA, USA). The non-robotic group comprised laparoscopic and open surgery (772 laparoscopic and 112 open procedures; *n* = 884), yielding 2139 patients (1255 robotic, 884 non-robotic). Because pooling structurally different comparators may obscure divergent effects, the primary comparison was robotic versus laparoscopic surgery in colorectal and uterine cancer. Robotic versus open surgery in these cancers and the four-cancer pooled comparison were secondary. The diagnostic-code groups included colon, rectal, and selected anal-canal cancers; cervical and corpus uterine cancers; and kidney and upper urinary tract cancers. Specific resection subtypes could not be distinguished in the available data, so cancer group alone does not establish comparable operations.

### 2.4. T Category and FIGO Stage Grouping

Disease extent was grouped according to the available tumor–node–metastasis (TNM) T category or International Federation of Gynecology and Obstetrics (FIGO) stage: T1 or FIGO I, T2 or FIGO II, and T3–T4 or FIGO III–IV. This grouping was used as a pragmatic summary of the available disease-extent information for covariate adjustment, with cancer type included separately in the models. These groups were used as an adjustment covariate and were not intended to represent equivalent prognostic stages across cancers. For TNM data, the grouping used the T component alone and did not incorporate nodal or metastatic status. The classification criteria are detailed in the Supplementary Methods.

Staging information was obtained from the electronic medical records. Although NX and MX were recoded to zero during preprocessing, nodal and metastatic components were not used in the final three-group classification; that recoding therefore did not determine group assignment. When staging information was unavailable, it was extracted from pathology reports using automated text analysis. All assignments were independently reviewed by two oncological surgeons, with discordant cases adjudicated by a third senior reviewer. This review checked individual assignments but did not validate equivalence between cancer staging systems. Pathological findings may have been obtained after surgery, and adjustment for these groups does not provide complete control for disease severity.

### 2.5. Outcomes

The primary outcome was LOS, from admission to discharge. Secondary outcomes were 30-day rehospitalization, 30-day all-cause mortality, postoperative complications, surgical site infection, and perioperative blood transfusion. Projected differences in index-admission bed-days per 100 procedures were estimated by multiplying the adjusted mean LOS difference by 100. Blood transfusion is reported descriptively. Rehospitalization was defined as readmission to the study hospital within 30 days after surgery. Planned and unplanned admissions were not distinguished, and the reasons for and duration of readmission were unavailable. Deaths occurring at the study hospital were identified from death dates in the electronic medical records, irrespective of cause. Patients without a death date were classified as having no mortality event. Mortality was defined as death within 30 days after surgery; deaths outside this hospital may not have been captured. Postoperative complications were identified by reviewing pathology test results in the electronic medical records. Surgical site infections were identified from entries in the electronic medical records. Clavien–Dindo grades were not assigned, and complication ascertainment did not include a systematic review of the complete medical record. Complication analyses were therefore considered exploratory.

### 2.6. Statistical Analysis

Baseline characteristics were summarized as means with standard deviations (SDs) or frequencies with percentages, and compared using t tests, chi-square tests, or Fisher exact tests as appropriate. For the restricted primary cohort, absolute standardized mean differences (SMDs) were calculated; category-specific SMDs compare each category with all others.

In the primary cohort and unweighted secondary analyses, outcomes were analyzed using generalized linear models, with cancer type entered as a fixed effect. There were only three surgical departments in the full cohort and two in the primary cohort; department random-intercept models were therefore evaluated as sensitivity analyses rather than used for primary inference. LOS was modeled using gamma regression with a log link (reported as LOS ratios) and binary outcomes using logistic regression (adjusted odds ratios, aORs, with 95% confidence intervals, CIs).

Primary models included surgical approach, age, sex, T category/FIGO stage group, and cancer type. Operative time and staffing variables (numbers of assisting physicians and nurses) were excluded from primary adjustment because they may be consequences of platform selection, and included only in sensitivity analysis. Constant covariates were omitted in cancer-specific models. Absolute differences in LOS and 30-day rehospitalization were estimated using G-computation: each model was applied to the relevant analysis population twice, with all patients assigned in turn to each platform, and the difference in marginal means computed. We interpreted these estimates as covariate-standardized associations, given the potential for residual confounding and the limited overlap in the four-cancer cohort. Differences were defined as comparator minus robotic. Both platform-specific means and their difference were calculated over the same analysis population. Bootstrap percentile 95% CIs were based on 1000 patient-level resamples stratified by cancer and observed approach. All 1232 primary patients were retained in the primary and same-sample sensitivity models. Tests were two-tailed and *p* < 0.05 was considered significant. Analyses used R version 4.3.0 (R Foundation for Statistical Computing, Vienna, Austria). Reporting follows the STROBE guideline [12].

### 2.7. Subgroup and Sensitivity Analyses

To assess sensitivity to comparator composition, secondary analyses compared robotic surgery against open surgery in colorectal and uterine cancer (462 robotic and 70 open patients) and against pooled non-robotic surgery in the four-cancer cohort. Cancer-specific estimates and cancer-by-platform interaction tests were calculated for LOS and rehospitalization; interaction tests used an F test for gamma models and a likelihood-ratio test for logistic models.

Sensitivity analyses added operative time and staffing to the primary model or added calendar year to the primary model; both analyses included the same 1232 patients. Department random-intercept fits were also attempted; the latent logistic intraclass correlation (ICC) was calculated as department variance divided by the sum of department variance and π^2^/3. The research team did not use surgeon identifiers for ethical and policy reasons; surgeon-level effects, volume, and learning curves were therefore not assessed. Calendar-year adjustment addresses variation over time but does not control for surgeon-specific experience or outcomes. The patient-level bootstrap likewise does not account for clustering within surgeons. Secondary sensitivity analyses in the four-cancer cohort compared generalized linear models with and without operative time (Table S1). Both specifications included age, sex, cancer type, T category/FIGO stage group, and staffing; only adjustment for operative time differed. Secondary analyses also used inverse probability weighting. For weighting, propensity scores for receipt of robotic surgery were estimated by logistic regression including age, sex, T category/FIGO stage group, and cancer type. Variables realized after the platform had been selected, including operative time and staffing, were excluded from the propensity score model. Stabilized inverse probability of treatment weights were applied with robust variance estimation, and the analysis was repeated with weights truncated at the 1st and 99th percentiles. Balance was assessed by weighted standardized mean differences, and the effective sample size was calculated to quantify the precision cost of weighting. For sparse binary outcomes, we assessed separation at the coefficient level and checked the numerical stability of finite surgical-approach estimates. Mortality and infection estimates were interpreted as exploratory.

## 3. Results

### 3.1. Baseline Characteristics

The primary cohort comprised 1232 patients with colorectal or uterine cancer: 462 underwent robotic and 770 underwent laparoscopic surgery (Table 1). Mean age was 57.5 (SD 12.3) versus 64.0 (SD 13.9) years (absolute SMD 0.49), and 61.3% versus 61.0% were female. Colorectal cancer accounted for 59.7% versus 66.8%, and T3–T4 or FIGO III–IV disease for 35.1% versus 50.1% (absolute SMD 0.31). Thus, restriction improved representation of both platforms within each cancer but did not eliminate differences in patient characteristics. Cancer-specific characteristics, including crude LOS, are shown in Table S5B.

The secondary four-cancer cohort included 2139 patients: 1255 in the robotic group and 884 in the laparoscopic and open group (Table S5A). Mean age was 64.19 (SD 10.66) versus 63.92 (SD 13.94) years (*p* = 0.614), and male patients were more frequent in the robotic group (76.9% versus 41.1%; *p* < 0.001). Cancer type distribution differed markedly (*p* < 0.001): prostate cancer accounted for 60.8% of robotic procedures, whereas colorectal cancer (62.6%) predominated in the comparator group. Among patients undergoing laparoscopic or robotic surgery, prostate and renal cancers contributed only one laparoscopic case each. The distribution of T category/FIGO stage groups also differed (*p* < 0.001). Mean operative time was 165.22 versus 155.38 min (*p* = 0.009).

### 3.2. Adjusted Surgical Outcomes

In the primary colorectal and uterine cancer cohort, robotic surgery was associated with a 22% lower adjusted mean LOS than laparoscopic surgery (LOS ratio 0.78, 95% CI 0.73–0.83; *p* < 0.001; Table 2). Higher odds were observed for 30-day rehospitalization (aOR 1.82, 95% CI 1.34–2.47; 289 events). Estimates for surgical site infection (aOR 0.29, 95% CI 0.08–1.06; 17 events), 30-day mortality (aOR 0.81, 95% CI 0.42–1.60; 50 events), and postoperative complications (aOR 0.75, 95% CI 0.37–1.49; 42 events) were imprecise.

Because prostate cancer was treated almost exclusively with the robotic platform, case-mix may account for a substantial portion of the full-cohort difference. Statistical adjustment may not fully disentangle this structural collinearity, and the secondary four-cancer pooled estimate should not be read as a platform-specific effect.

### 3.3. Analyses by Comparator

Estimates differed by comparator and cancer type (Table 2). Against open surgery in colorectal and uterine cancer, robotic surgery was associated with shorter LOS (LOS ratio 0.50, 95% CI 0.44–0.57); the rehospitalization estimate was imprecise (aOR 0.88, 95% CI 0.48–1.60). The secondary four-cancer pooled estimates were 0.75 (95% CI 0.71–0.80) for LOS and 1.36 (95% CI 1.02–1.82) for rehospitalization.

Crude rates also diverged by malignancy: among patients undergoing laparoscopic or robotic surgery, rehospitalization was higher with robotic surgery in colorectal cancer (44.2% versus 25.7%) and lower in uterine cancer (3.8% versus 10.9%; Table S4). Because prostate and renal cancers contributed only one laparoscopic case each, this comparison principally reflects colorectal and uterine cancer. The infection model against open surgery was not estimable because no infection events occurred in the open group. Cancer-specific adjusted rehospitalization odds ratios were 2.50 (95% CI 1.77–3.53) for colorectal and 0.38 (95% CI 0.16–0.90) for uterine cancer (interaction *p* < 0.001). LOS ratios were 0.86 (95% CI 0.79–0.93) and 0.67 (95% CI 0.60–0.75), respectively (interaction *p* = 0.002; Table S6). These exploratory interaction tests were not adjusted for multiple testing.

### 3.4. Sensitivity Analyses

Adding operative time and staffing to the primary model changed the LOS ratio from 0.78 to 0.75 (95% CI 0.70–0.80) and the rehospitalization aOR from 1.82 to 1.64 (95% CI 1.19–2.25). Calendar-year adjustment yielded 0.78 (95% CI 0.73–0.84) and 1.83 (95% CI 1.35–2.49), respectively (Table S7). All models retained the same 1232 patients. Department variance estimates were zero or numerically near zero, with latent logistic ICCs of zero or approximately 8.15 × 10^−21^. In the primary cohort, all ten department random-intercept models had boundary variance estimates or numerical estimation problems; their surgical-approach estimates are therefore not presented (Table S8). These findings do not establish the absence of clustering. Sensitivity analyses with and without adjustment for operative time in the secondary four-cancer cohort are presented in Table S1.

In the secondary four-cancer analysis, after stabilized inverse probability weighting, the maximum absolute standardized mean difference fell from 1.51 to 0.10 (Table S2, Figure S1), at the cost of an effective sample size of 817 of 2139 patients (38.2%; Figure S2). The LOS association persisted (LOS ratio 0.79, 95% CI 0.75–0.84), whereas mortality (0.61, 95% CI 0.29–1.27) and infection (0.37, 95% CI 0.10–1.39) estimates were imprecise (Table S3). Truncation did not materially change results. The weighted rehospitalization estimate (aOR 0.87, 95% CI 0.58–1.29) differed in direction from the covariate-adjusted estimate (aOR 1.36, 95% CI 1.02–1.82; Table S3). Limited overlap and differences between the analytical approaches preclude attributing this discrepancy to a single cause. These secondary weighting results do not overcome the lack of within-cancer overlap and are not used as primary evidence of a platform-specific association.

### 3.5. Standardized Outcomes and Projected Bed-Days

In the primary cohort, standardized mean LOS was 8.29 days for robotic and 10.63 days for laparoscopic surgery (difference 2.34 days; 95% CI 1.80–2.93; Table 3). Standardized 30-day rehospitalization was 29.56% versus 20.11% (laparoscopic minus robotic, −9.45 percentage points; 95% CI −14.08 to −4.66), consistent in direction with crude rates of 27.9% versus 20.8%. The adjusted LOS difference corresponds to a projected difference of 234 index-admission bed-days per 100 procedures, a projection contingent on the case-mix and care setting of this cohort. In the secondary four-cancer analysis, crude rehospitalization was 10.9% robotic versus 22.4% non-robotic, whereas standardized risks were 17.73% versus 14.44% (difference −3.29 percentage points; 95% CI −6.39 to −0.22; Table S9). The robotic group included 763 prostate cases with only 7 readmissions, whereas the non-robotic group included 38 prostate cases. Standardization changes this case-mix contribution and reverses the crude direction; limited overlap makes this result vulnerable to extrapolation.

## 4. Discussion

The primary LOS association in colorectal and uterine cancer persisted in sensitivity analyses adjusting for operative time and staffing or calendar year. Mortality and infection estimates were imprecise and did not establish a perioperative safety benefit. Randomized and pooled evidence suggests that comparisons of robotic and laparoscopic surgery depend on the procedure and outcome. Our findings also highlight the need to evaluate post-discharge hospital use when interpreting shorter index admissions.

The primary and sensitivity analyses yielded LOS ratios of 0.75–0.78. Differences in procedure type, complexity, surgeon-related factors, learning curves, and institutional perioperative practices may have contributed to this association. Absolute stays here exceed those typically reported from Western centers. LOS was measured from admission, not surgery, and includes preoperative days. Robotic mean stays were 10.42 days for colorectal, 4.53 for uterine, and 7.57 for prostate cancer. Reimbursement arrangements, discharge practices, rehabilitation, and home support may contribute, but their effects were not measured. The 234-bed-day projection represents the difference in index-admission LOS multiplied by 100, rather than observed beds released by changing surgical practice. It should not be transferred directly to Western enhanced-recovery settings with shorter baseline stays. Relative associations may also vary across care pathways, and readmission days were not deducted.

Previous comparative studies have reported lower blood loss and shorter hospitalization with minimally invasive approaches than with open surgery [10,13,14]. Comparisons between robotic and laparoscopic surgery have shown procedure-specific differences in short-term outcomes [4,7,8,9,15,16]. ROLARR found no significant reduction in conversion or complications with robotic rectal resection [7,15]. In urologic oncology, robotic approaches compare favorably with open but less clearly with laparoscopic surgery [10,14]. Meta-analyses of partial nephrectomy have reported advantages of robotic surgery for selected perioperative outcomes, with similar results for other outcomes [16,17]. Reported differences depend on the operation, comparator, and outcome. The REAL trial reported favorable short-term outcomes for robotic versus laparoscopic middle and low rectal resection [18]. LAP-01 reported better early continence recovery after robotic versus conventional laparoscopic radical prostatectomy [19]. RAZOR established noninferiority of robotic versus open radical cystectomy for 2-year progression-free survival [20]. The Yaxley and Coughlin randomized reports provide functional and oncological outcome comparisons for robotic versus open radical prostatectomy [21,22]. These trials support procedure- and comparator-specific interpretation; their findings cannot be directly generalized to our heterogeneous cancer groups, and bladder cancer was not included in this cohort.

Mortality, infection, and complication estimates were imprecise. Only 17 surgical site infections occurred, and the primary mortality and infection confidence intervals included the null. Complications were identified through review of pathology test results without systematic review of the complete medical record or assignment of Clavien–Dindo grades. This method may miss complications not identified through such testing. The low rates therefore do not provide a comprehensive estimate of postoperative morbidity and cannot establish the relative safety of the surgical approaches.

A critical consideration is the heterogeneity of the comparator group, which reflects real-world practice but complicates interpretation. In the primary laparoscopic comparison, rehospitalization associations differed in direction between colorectal and uterine cancer; the secondary open comparison was imprecise. The LOS association versus open surgery may partly reflect minimally invasive access, which laparoscopy shares; the robotic-versus-laparoscopic comparison does not by itself isolate a causal platform-specific contribution. A pooled conventional comparator, though common, can cancel opposing signals and yield an oversimplified conclusion.

In the primary cohort, higher odds of rehospitalization were observed both without and with adjustment for operative time and staffing (aORs, 1.82 and 1.64, respectively; Table S7). In the secondary four-cancer pooled comparison, the aOR was 1.36 in the covariate-adjusted model and 0.87 with stabilized weighting (Table S3). These differences should be interpreted in the context of the marked imbalance in cancer type and the opposing cancer-specific associations. With follow-up ending 30 days after surgery, patients discharged earlier after surgery have more time for a readmission to occur within this period. The available data cannot determine why rehospitalization was frequent in the colorectal cohort because planned and unplanned admissions were not distinguished and the reasons for and duration of readmission were unavailable. Hospital stay and readmission have been evaluated together in other surgical populations [23,24]. In particular, the 44.2% crude rehospitalization rate after robotic colorectal surgery should not be interpreted as a postoperative complication rate. The data cannot determine whether shorter index admissions were offset by subsequent hospital use. Prospective studies that distinguish planned from unplanned readmissions and measure their duration are therefore required.

We examined the same outcomes using separate laparoscopic and open comparators. G-computation expresses these associations as absolute differences alongside relative estimates [25]. The bed-day projection alone cannot establish the economic value of robotic surgery, which also depends on acquisition and maintenance costs, operative time, and subsequent hospital use [11,26,27].

### Limitations

This study has several limitations. Residual confounding may persist because the T category/FIGO stage groups are not equivalent prognostic stages across cancers and the TNM-based groups used the T component without nodal or metastatic status. Specific resection subtypes and procedural complexity could not be distinguished, so differences in the operations performed may have contributed to the observed associations despite restriction by cancer type. In the secondary four-cancer analysis, weighting improved measured balance but reduced the effective sample size to 817 of 2139. The near-exclusive use of robotic surgery for prostate cancer produced collinearity among surgical approach, cancer type, and sex that adjustment could not resolve [28]. Only two departments contributed to the primary cohort. Surgeon-level clustering, volume, and learning curves were not assessed; these factors may confound associations, and confidence intervals may be too narrow even after calendar-year adjustment. The extent and direction of these biases are uncertain. Mortality ascertainment was limited to deaths occurring at the study hospital within 30 days after surgery. Complication ascertainment through pathology test results may underestimate postoperative morbidity. Infection, mortality, and complication estimates were limited by small event counts, and some subgroup models were not estimable because of separation. Rehospitalization was identified from administrative records, and planned and unplanned admissions were not distinguished. Operative time and staffing may be mediators and were included only in sensitivity analysis. Blood transfusion was reported descriptively without adjusted comparisons. Long-term oncologic outcomes were not assessed, although survival may differ by surgical approach in some settings [29,30]. Cost-effectiveness was not assessed [11,26,27]. Finally, findings from this single center may not apply to settings with different admission and discharge practices.

## 5. Conclusions

The observed LOS difference should be interpreted alongside the cancer-specific rehospitalization patterns. The adjusted LOS difference corresponded to a setting-specific projection of 234 index-admission bed-days per 100 procedures. Rehospitalization was more frequent after robotic surgery for colorectal cancer and less frequent for uterine cancer; planned and unplanned admissions were not distinguished. Estimates for mortality, complications, and infection were inconclusive. Residual confounding by disease severity, operation subtype, and surgeon-related factors limits causal interpretation. The bed-day projection does not establish net savings in hospital use, which requires assessment of subsequent admissions.

## Figures and Tables

**Table 1:** Characteristics and crude outcomes of the primary colorectal and uterine cancer cohort, by surgical approach.

Characteristic	Robotic (*n* = 462)	Laparoscopic (*n* = 770)	Absolute SMD
Age, years	57.5 (12.3)	64.0 (13.9)	0.49
Female sex	283 (61.3)	470 (61.0)	0.00
Male sex	179 (38.7)	300 (39.0)	0.00
Colorectal cancer	276 (59.7)	514 (66.8)	0.15
Uterine cancer	186 (40.3)	256 (33.2)	0.15
T1 or FIGO I	197 (42.6)	245 (31.8)	0.23
T2 or FIGO II	103 (22.3)	139 (18.1)	0.11
T3–T4 or FIGO III–IV	162 (35.1)	386 (50.1)	0.31
Operative time, min	190.6 (121.3)	154.2 (88.7)	0.34
Assisting physicians, *n*	0.8 (0.7)	0.6 (0.7)	0.16
Nurses, *n*	2.9 (1.2)	2.9 (1.2)	0.04
Length of stay, days	8.0 (4.9)	10.8 (7.4)	—
30-day rehospitalization	129 (27.9)	160 (20.8)	—
30-day mortality	13 (2.8)	37 (4.8)	—
Postoperative complications	13 (2.8)	29 (3.8)	—
Surgical site infection	3 (0.6)	14 (1.8)	—

Values are mean (SD) or *n* (%). SMD denotes absolute standardized mean difference; category-specific SMDs compare each category with all others. No demographic, operative-time, or staffing values were missing. T category/FIGO stage groups do not represent equivalent prognostic stages across cancers (Section 2.4). Rehospitalization and mortality were assessed within 30 days after surgery. SD, standard deviation. Complications were identified from pathology test results and were analyzed as an exploratory outcome.

**Table 2:** Adjusted associations between robotic surgery and perioperative outcomes, by comparator and analysis population.

Outcome	Events in Primary Cohort	Primary vs. Laparoscopic (*n* = 1232)	Secondary vs. Open (*n* = 532)	Secondary Four-Cancer Pooled (*n* = 2139)
Length of stay	–	0.78 (0.73–0.83)	0.50 (0.44–0.57)	0.75 (0.71–0.80)
30-day rehospitalization	289	1.82 (1.34–2.47)	0.88 (0.48–1.60)	1.36 (1.02–1.82)
30-day mortality	50	0.81 (0.42–1.60)	0.17 (0.07–0.41)	0.65 (0.35–1.21)
Postoperative complications	42	0.75 (0.37–1.49)	0.48 (0.12–1.98)	0.82 (0.43–1.57)
Surgical site infection	17	0.29 (0.08–1.06)	Not estimable	0.31 (0.09–1.13)

Values are length-of-stay ratios (for length of stay) or adjusted odds ratios (all other outcomes) with 95% confidence intervals, obtained from generalized linear models adjusted for age, sex, cancer type, and T category/FIGO stage group, without operative time or staffing. Events refer to the primary colorectal and uterine robotic-versus-laparoscopic cohort (*n* = 1232). The open comparison includes only these two cancers (*n* = 532); the pooled non-robotic comparison includes all four cancers (*n* = 2139) and is secondary. The infection model versus open surgery was not estimable owing to separation (no infection events occurred in the open group). Mortality and infection estimates are exploratory. Complications were identified from pathology test results and were analyzed as an exploratory outcome.

**Table 3:** Standardized length of stay and 30-day rehospitalization, with the projected difference in index-admission bed-days (primary colorectal and uterine cohort, robotic versus laparoscopic surgery).

Outcome	Robotic	Laparoscopic	Laparoscopic Minus Robotic (95% CI)
Standardized mean length of stay, days	8.29	10.63	2.34 (1.80 to 2.93)
Standardized 30-day rehospitalization, %	29.56	20.11	−9.45 (−14.08 to −4.66)
Projected difference in index-admission bed-days per 100 procedures	–	–	234 (180 to 293)

Estimates were obtained by G-computation applied to the fitted primary generalized linear models. Both platform-specific means were standardized over the same 1232 patients, and differences were calculated as laparoscopic minus robotic. Percentile confidence intervals used 1000 patient-level bootstrap resamples stratified by cancer and observed approach. Bed-days per 100 procedures equal 100 times the adjusted admission LOS difference; this is a setting-specific projection, not an estimate of causal or net savings. Readmission duration was not available and surgeon-level clustering was not included in the bootstrap. Rehospitalization differences are expressed in percentage points. –, not applicable; CI, confidence interval.

## Data Availability

The data presented in this study are available on request from the corresponding author. The data are not publicly available because they contain information that could compromise the privacy of research participants and because their release is restricted by the institutional data governance policy of Keimyung University Dongsan Hospital.

## Supplementary Materials

The following supporting information can be downloaded at: https://www.mdpi.com/article/10.3390/jcm15207846/s1, Supplementary Methods: Sequential exclusion criteria and T category and FIGO stage grouping; Table S1: Sensitivity analysis: models with and without adjustment for operative time (secondary four-cancer analysis); Table S2: Covariate balance and weight diagnostics for inverse probability weighting (secondary four-cancer analysis); Table S3: Covariate-adjusted and inverse probability weighted estimates (robotic versus pooled non-robotic surgery; secondary four-cancer analysis); Table S4: Crude 30-day rehospitalization by cancer type and surgical approach; Figure S1: Covariate balance before and after inverse probability weighting; Figure S2: Distribution of stabilized inverse probability weights; Table S5A: Characteristics and crude outcomes of the secondary four-cancer cohort; Table S5B: Primary cohort characteristics by cancer and surgical approach; Table S6: Cancer-specific adjusted associations, robotic versus laparoscopic surgery; Table S7: Primary cohort sensitivity models; Table S8: Department counts and random-intercept sensitivity models; Table S9: Standardized outcomes in the secondary four-cancer analysis; STROBE Statement—Checklist of Items for Cohort Studies.
