# Supplementary material for: Postoperative Outcomes of Robotic Versus Conventional Surgery in a Multi-Cancer Cohort: A Retrospective Cohort Study
Source: J Clin Med. 2026 Oct 12;15(20):7846. doi: 10.3390/jcm15207846 (PMC13705545; doi:10.3390/jcm15207846)
Supplement: Supplementary file 1 [file jcm-15-07846-s001.zip › STROBE_Checklist.pdf]

STROBE Statement—Checklist of Items for Cohort Studies

Postoperative Outcomes of Robotic Versus Conventional Surgery in a Multi-Cancer Cohort: A Retrospective Cohort Study

Submitted to Journal of Clinical Medicine (MDPI) as an Article.

Item	Section	Recommendation	Location in manuscript
1	Title and abstract	(a) Indicate the study design with a commonly used term in the title or the abstract. (b) Provide in the abstract an informative and balanced summary of what was done and what was found.	Title; Abstract (Methods, Results)
2	Introduction — Background/rationale	Explain the scientific background and rationale for the investigation being reported.	Section 1, paragraphs 1–2
3	Introduction — Objectives	State specific objectives, including any prespecified hypotheses.	Abstract (Background/Objectives); Section 1, paragraph 3
4	Methods — Study design	Present key elements of study design early in the paper.	Section 2.1
5	Methods — Setting	Describe the setting, locations, and relevant dates, including periods of recruitment, exposure, follow-up, and data collection.	Sections 2.1–2.2 and 2.5; Rehospitalization and mortality are defined within 30 days after surgery.
6	Methods — Participants	(a) Give the eligibility criteria, and the sources and methods of selection of participants. Describe methods of follow-up. (b) For matched studies, give matching criteria and number of exposed and unexposed.	Sections 2.2–2.3; Supplementary Methods S1; specific resection subtypes could not be distinguished. Matching: not applicable.
7	Methods — Variables	Clearly define all outcomes, exposures, predictors, potential confounders, and effect modifiers. Give diagnostic criteria, if applicable.	Sections 2.3–2.5; Supplementary Methods S2.
8	Methods — Data sources/measurement	For each variable of interest, give sources of data and details of methods of assessment. Describe comparability of assessment methods if there is more than one group.	Sections 2.4–2.5; Supplementary Methods S2.
9	Methods — Bias	Describe any efforts to address potential sources of bias.	Sections 2.6 and 2.7
10	Methods — Study size	Explain how the study size was arrived at.	Section 2.2; Supplementary Methods S1
11	Methods — Quantitative variables	Explain how quantitative variables were handled in the analyses. If applicable, describe which groupings were chosen and why.	Sections 2.4 and 2.6
12	Methods — Statistical methods	(a) Describe all statistical methods, including those used to control for confounding. (b) Describe any methods used to examine subgroups and interactions. (c) Explain how missing data were addressed. (d) If applicable, explain how loss to follow-up was addressed. (e) Describe any sensitivity analyses.	Sections 2.5–2.7 and 4.1; Tables S1–S3 and S7–S8. S1 and the covariate-adjusted S3 column use generalized linear models without a department random intercept. Hospital-based outcome ascertainment and sparse-event limitations are described in Sections 2.5, 2.7 and 4.1.

Item	Section	Recommendation	Location in manuscript
13	Results — Participants	(a) Report numbers of individuals at each stage of study. (b) Give reasons for non-participation at each stage. (c) Consider use of a flow diagram.	Sections 2.2 and 3.1; Supplementary Methods S1. Exclusion criteria and final cohort counts are reported; intermediate exclusion counts are not reported.
14	Results — Descriptive data	(a) Give characteristics of study participants and information on exposures and potential confounders. (b) Indicate number of participants with missing data for each variable of interest. (c) Summarise follow-up time (e.g., average and total amount).	Section 3.1; Table 1 (primary cohort); Table S5A (secondary full cohort) and S5B (primary cohort by cancer and approach, including crude LOS). The 30-day period for rehospitalization and mortality begins on the date of surgery (Section 2.5).
15	Results — Outcome data	Report numbers of outcome events or summary measures over time.	Sections 3.2–3.5; Tables 2–3 and S4–S9; mortality ascertainment limitations in Section 4.1.
16	Results — Main results	(a) Give unadjusted estimates and, if applicable, confounder-adjusted estimates and their precision. Make clear which confounders were adjusted for and why they were included. (b) Report category boundaries when continuous variables were categorized. (c) If relevant, consider translating estimates of relative risk into absolute risk.	Sections 3.2–3.5; Tables 2–3 and S6–S9.
17	Results — Other analyses	Report other analyses done, such as analyses of subgroups and interactions, and sensitivity analyses.	Sections 3.3–3.4; Tables S1–S3 and S6–S8.
18	Discussion — Key results	Summarise key results with reference to study objectives.	Section 4, paragraph 1
19	Discussion — Limitations	Discuss limitations of the study, taking into account sources of potential bias or imprecision. Discuss both direction and magnitude of any potential bias.	Section 4.1; limitations of disease-extent adjustment, procedure type and complexity, surgeon-related factors, outcome ascertainment, and sparse-event inference are stated explicitly.
20	Discussion — Interpretation	Give a cautious overall interpretation of results considering objectives, limitations, multiplicity of analyses, results from similar studies, and other relevant evidence.	Section 4, paragraphs 2–7; Section 5
21	Discussion — Generalisability	Discuss the generalisability (external validity) of the study results.	Section 4, including local admission and discharge practices; Sections 3.5 and 5. Bed-day projections concern index admissions in this setting and do not establish actual or net savings.
22	Other — Funding	Give the source of funding and the role of the funders for the present study and, if applicable, for the original study on which the present article is based.	Funding; Conflicts of Interest

Section numbers refer to the numbered sections of the submitted manuscript. Tables S1–S9 and Figures S1–S2 refer to the Supplementary Materials. An explanation and elaboration article discusses each checklist item and gives methodological background and published examples of transparent reporting; it is available together with the STROBE checklist at https://www.strobe-statement.org.
