# Supplementary material for: Postoperative Outcomes of Robotic Versus Conventional Surgery in a Multi-Cancer Cohort: A Retrospective Cohort Study
Source: J Clin Med. 2026 Oct 12;15(20):7846. doi: 10.3390/jcm15207846 (PMC13705545; doi:10.3390/jcm15207846)
Supplement: Supplementary file 1 [file jcm-15-07846-s001.zip › Supplementary_Materials.pdf]

# Supplementary Materials

*Postoperative Outcomes of Robotic Versus Conventional Surgery in a Multi-Cancer Cohort: A Retrospective Cohort Study*

Jaehyeong Cho, Hyoshuk Kim and Chi-Heum Cho

## Contents

Supplementary Methods. Sequential exclusion criteria and T category and FIGO stage grouping

Table S1. Sensitivity analysis: models with and without adjustment for operative time (secondary four-cancer analysis)

Table S2. Covariate balance and weight diagnostics for inverse probability weighting (secondary four-cancer analysis)

Table S3. Covariate-adjusted and inverse probability weighted estimates (secondary four-cancer analysis)

Table S4. Crude 30-day rehospitalization by cancer type and surgical approach

Figure S1. Covariate balance before and after inverse probability weighting (secondary four-cancer analysis)

Figure S2. Distribution of stabilized inverse probability weights (secondary four-cancer analysis)

Table S5. Additional cohort characteristics (S5A: secondary four-cancer cohort; S5B: primary cohort by cancer and approach)

Table S6. Cancer-specific adjusted associations, robotic versus laparoscopic surgery

Table S7. Primary cohort sensitivity models

Table S8. Department counts and random-intercept sensitivity models

Table S9. Standardized outcomes in the secondary four-cancer analysis

## Supplementary Methods

### S1. Sequential Exclusion Criteria

Patients meeting the inclusion criteria were excluded sequentially for the following reasons:

1. Concurrent diagnosis with more than one of the four index cancer types, as determined by overlapping ICD-10 codes.
2. Ambiguous surgical approach classification, defined as the coexistence of robotic and laparoscopic procedure codes within the same operative record, precluding unambiguous group assignment.
3. Duplicate administrative identifiers in the billing linkage file, indicating a data integrity issue.
4. Missing diagnosis code after record linkage.
5. Unavailable T category/FIGO stage group after structured-data and pathology extraction.
6. Irreconcilable inconsistency between admission, operative, and discharge records identified during data quality review (n = 2).

After sequential exclusions, 2,139 patients were retained for the final analysis (1,255 robotic, 772 laparoscopic, 112 open).

### S2. T Category and FIGO Stage Grouping

Disease extent was grouped according to the available tumor–node–metastasis (TNM) T category or International Federation of Gynecology and Obstetrics (FIGO) stage: T1 or FIGO I, T2 or FIGO II, and T3–T4 or FIGO III–IV. These groups were used as an adjustment covariate and were not intended to represent equivalent prognostic stages across cancers. For TNM data, the grouping used the T component alone and did not incorporate nodal or metastatic status. The rules used for group assignment are shown below.

| T category/FIGO stage group | Structured-data rule | Pathology extraction rule | Interpretation |
|---|---|---|---|
| T1 or FIGO I | FIGO I or T1 | FIGO I or T1 | Study-specific adjustment group |

| T category/FIGO stage group | Structured-data rule | Pathology extraction rule | Interpretation |
|---|---|---|---|
| T2 or FIGO II | FIGO II or T2 | FIGO II or T2 | Study-specific adjustment group |
| T3–T4 or FIGO III–IV | FIGO III–IV or T3–T4 | FIGO III–IV or T3–T4 | Study-specific adjustment group |

Staging information was obtained from the electronic medical records. Although NX and MX were recoded to zero during preprocessing, nodal and metastatic components were not used in the final three-group classification; that recoding therefore did not determine group assignment. When staging information was unavailable, it was extracted from pathology reports using automated text analysis. All assignments were independently reviewed by two oncological surgeons, with discordant cases adjudicated by a third senior reviewer. This review checked individual assignments but did not validate equivalence between cancer staging systems. Pathological findings may have been obtained after surgery, and adjustment for these groups does not provide complete control for disease severity.

**Table S1.** Sensitivity analysis: models with and without adjustment for operative time (secondary four-cancer analysis).

| Comparison | Outcome | With operative time | Without operative time | Change (%) |
|---|---|---|---|---|
| vs. Laparoscopic | Length of stay | 0.73 (0.69–0.77) | 0.77 (0.72–0.82) | +5.5 |
| | 30-day rehospitalization | 1.62 (1.18–2.23) | 1.82 (1.34–2.48) | +12.6 |
| | 30-day mortality | 0.53 (0.26–1.11) | 0.72 (0.36–1.44) | +34.9 |
| | Postoperative complications | 0.63 (0.31–1.31) | 0.78 (0.39–1.55) | +23.0 |
| | Surgical site infection | 0.24 (0.06–0.92) | 0.28 (0.08–1.03) | +18.1 |
| vs. Open | Length of stay | 0.59 (0.54–0.65) | 0.61 (0.55–0.67) | +2.4 |
| | 30-day rehospitalization | 0.35 (0.19–0.63) | 0.44 (0.25–0.78) | +25.9 |
| | 30-day mortality | 0.20 (0.09–0.45) | 0.26 (0.12–0.57) | +30.2 |
| | Postoperative complications | 0.57 (0.15–2.16) | 0.74 (0.20–2.72) | +28.0 |
| | Surgical site infection | Not estimable | Not estimable | – |
| vs. Non-robotic pooled | Length of stay | 0.72 (0.68–0.76) | 0.75 (0.71–0.80) | +4.9 |
| | 30-day rehospitalization | 1.18 (0.87–1.60) | 1.36 (1.02–1.82) | +15.4 |
| | 30-day mortality | 0.50 (0.26–0.94) | 0.64 (0.34–1.18) | +28.2 |
| | Postoperative complications | 0.67 (0.34–1.32) | 0.82 (0.43–1.57) | +22.6 |
| | Surgical site infection | 0.26 (0.07–0.99) | 0.30 (0.08–1.10) | +16.9 |

Values are length-of-stay ratios (for length of stay) or adjusted odds ratios (all other outcomes) with 95% confidence intervals, obtained from generalized linear models without a department random intercept; gamma models with a log link were used for LOS and logistic models for binary outcomes. Both specifications include age, sex, cancer type, T category/FIGO stage group, and staffing variables; only adjustment for operative time differs. Paired models used the same patients. Change (%) equals 100 × (estimate without operative time − estimate with operative time) / estimate with operative time, calculated before rounding. The infection model comparing robotic with open surgery had no finite surgical-approach estimate in either specification because no infections occurred in the open group; –, not applicable. These analyses use the secondary four-cancer cohort; sensitivity analyses in the primary colorectal and uterine cancer cohort are presented in Table S7. No deaths occurred in the renal group, and no infections occurred in the renal or prostate groups. Separation affected these cancer-type coefficients. In the mortality and infection models with finite surgical-approach estimates, these estimates were numerically stable across convergence tolerances; their Wald intervals remain exploratory. Complications were identified from pathology test results and were analyzed as an exploratory outcome.

**Table S2.** Covariate balance and weight diagnostics for inverse probability weighting (secondary four-cancer analysis).

| Variable | Unweighted | Stabilized IPTW | Truncated IPTW |
|---|---|---|---|
| Age | 0.022 | 0.055 | 0.040 |

| Variable | Unweighted | Stabilized IPTW | Truncated IPTW |
|---|---|---|---|
| Sex (male) | 0.782 | 0.022 | 0.072 |
| T1 or FIGO I | −0.206 | −0.050 | −0.053 |
| T2 or FIGO II | 0.324 | 0.053 | 0.132 |
| T3–T4 or FIGO III–IV | −0.103 | −0.004 | −0.067 |
| Cancer type: colorectal | −0.901 | 0.007 | −0.060 |
| Cancer type: uterine | −0.425 | 0.012 | −0.033 |
| Cancer type: prostate | 1.511 | 0.015 | 0.110 |
| Cancer type: renal | 0.140 | −0.099 | −0.065 |
| Maximum absolute SMD | 1.511 | 0.099 | 0.132 |
| Effective sample size, n (%) | 2,139 (100) | 817 (38.2) | 1,044 (48.8) |
| Mean weight | 1.00 | 1.00 | 0.97 |
| Maximum weight | – | 15.8 | 7.82 |
| Patients with weight > 10, n | – | 13 | 0 |

Values are standardized mean differences (SMD) unless otherwise indicated. Propensity scores were estimated by logistic regression including age, sex, T category/FIGO stage group, and cancer type. Variables realized after the surgical platform had been selected were excluded from the propensity score model. Truncation was applied at the 1st and 99th percentiles of the stabilized weight distribution. Effective sample size was calculated using the Kish formula. The unweighted analysis assigns unit weights (mean 1.00). Weight diagnostics that do not apply to the unweighted analysis are indicated by a dash (–). IPTW, inverse probability of treatment weighting.

**Table S3.** Covariate-adjusted and inverse probability weighted estimates (robotic versus pooled non-robotic surgery; secondary four-cancer analysis).

| Outcome | Covariate-adjusted GLM | Stabilized IPTW | Truncated IPTW |
|---|---|---|---|
| Length of stay | 0.75 (0.71–0.80) * | 0.79 (0.75–0.84) * | 0.79 (0.74–0.83) * |
| 30-day rehospitalization | 1.36 (1.02–1.82) * | 0.87 (0.58–1.29) | 0.91 (0.65–1.27) |
| 30-day mortality | 0.65 (0.35–1.21) | 0.61 (0.29–1.27) | 0.59 (0.30–1.17) |
| Postoperative complications | 0.82 (0.43–1.57) | 0.91 (0.50–1.67) | 0.84 (0.46–1.53) |
| Surgical site infection | 0.31 (0.09–1.13) | 0.37 (0.10–1.39) | 0.34 (0.09–1.28) |

Values are length-of-stay ratios (for length of stay) or odds ratios (all other outcomes) with 95% confidence intervals. Weighted models were fitted with robust (sandwich) variance estimation. Covariate-adjusted estimates were obtained from generalized linear models including surgical approach, age, sex, cancer type, and T category/FIGO stage group, without operative time, staffing, or a department random intercept. These models match the secondary four-cancer pooled models in Table 2. Weighted estimates were obtained using stabilized or truncated inverse probability weights. In the covariate-adjusted mortality and infection models, separation affected cancer-type coefficients; the surgical-approach estimates were finite and numerically stable. These sparse-event Wald intervals are exploratory. * $p < 0.05$. GLM, generalized linear model; IPTW, inverse probability of treatment weighting. Complications were identified from pathology test results and were analyzed as an exploratory outcome.

**Table S4.** Crude 30-day rehospitalization by cancer type and surgical approach.

| Cancer type | Laparoscopic, n | Rehospitalized, n (%) | Robotic, n | Rehospitalized, n (%) |
|---|---|---|---|---|
| Colorectal | 514 | 132 (25.7) | 276 | 122 (44.2) |
| Uterine | 256 | 28 (10.9) | 186 | 7 (3.8) |
| Prostate | 1 | 0 (0) | 763 | 7 (0.9) |
| Renal | 1 | 0 (0) | 30 | 1 (3.3) |
| Total | 772 | 160 (20.7) | 1,255 | 137 (10.9) |

Unadjusted counts restricted to patients undergoing laparoscopic or robotic surgery; open procedures are not shown. Prostate and renal cancers contributed only one laparoscopic case each, so the robotic-versus-laparoscopic comparison principally reflects colorectal and uterine cancer. Crude rehospitalization was higher with robotic surgery in colorectal cancer and lower in uterine cancer, that is, in opposite directions.

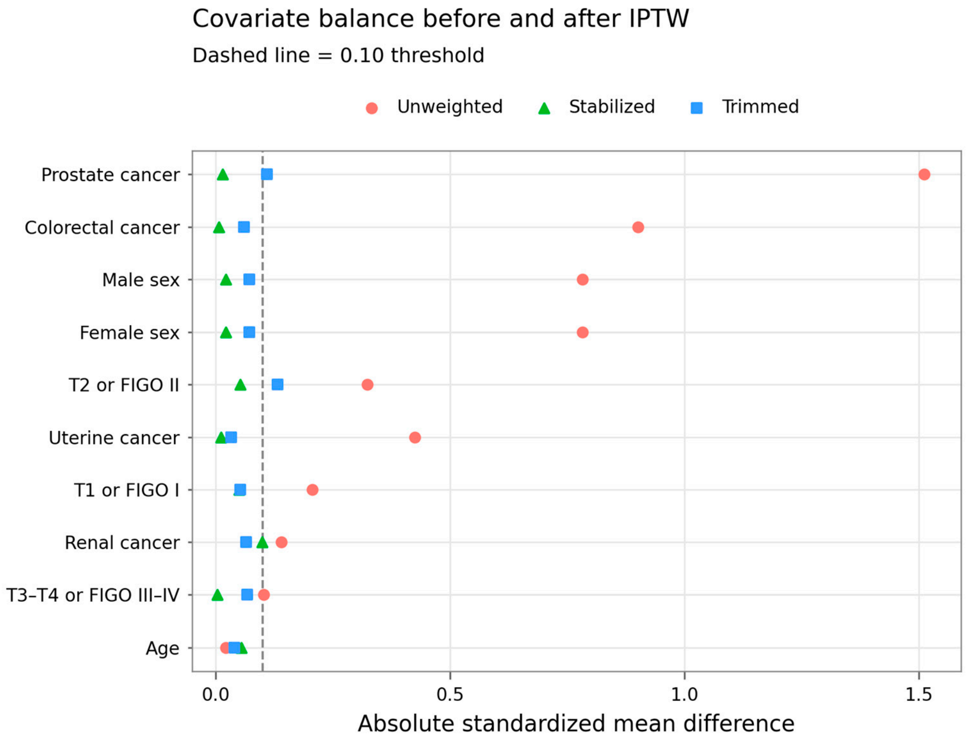


**Figure S1.** Covariate balance before and after inverse probability weighting. Absolute standardized mean differences are shown for each covariate before weighting (unweighted), after stabilized inverse probability weighting (stabilized), and after truncation at the 1st and 99th percentiles (labeled "Trimmed" in the figure). The dashed vertical line marks the conventional 0.10 threshold. The largest imbalance before weighting was in cancer type, reflecting the near-exclusive use of the robotic platform for prostate cancer; the maximum absolute standardized mean difference fell from 1.51 to 0.10 after stabilized weighting. (secondary four-cancer analysis)

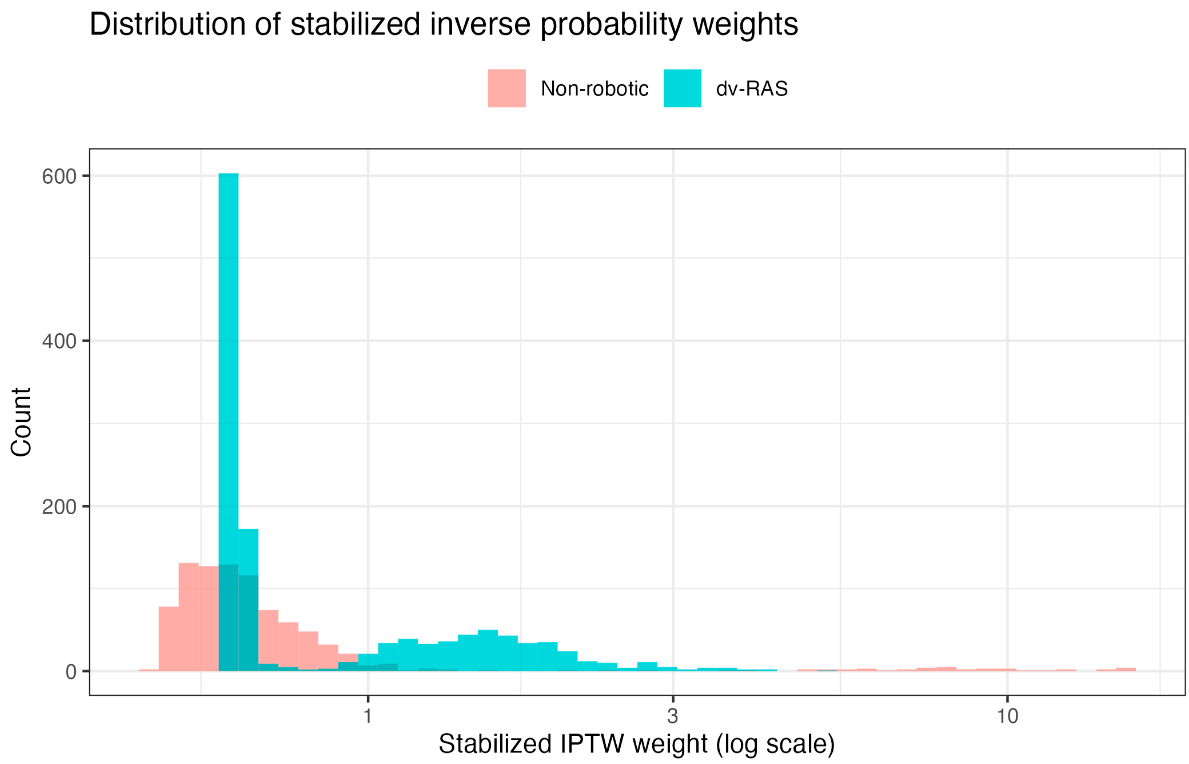


**Figure S2.** Distribution of stabilized inverse probability weights. The histogram shows stabilized weights by treatment group, plotted on a logarithmic scale; "dv-RAS" denotes robotic surgery. Thirteen patients received a weight exceeding 10 and the maximum stabilized weight was 15.8; these lie almost entirely in the non-robotic group, reflecting patients whose propensity for robotic surgery approached unity. The corresponding effective sample size was 817 of 2,139 patients, quantifying the precision cost of weighting in a cohort with limited overlap in surgical approach within individual malignancies. (secondary four-cancer analysis)

**Table S5A.** Characteristics and crude outcomes of the secondary four-cancer cohort.

| Characteristic | Laparoscopic and open (n = 884) | Robotic (n = 1,255) | p Value |
|---|---|---|---|
| Age, mean (SD), years | 63.92 (13.94) | 64.19 (10.66) | 0.614 |
| Sex, male, n (%) | 363 (41.1) | 965 (76.9) | <0.001 |
| Assisting physicians, mean (SD) | 0.65 (0.72) | 0.49 (0.64) | <0.001 |
| Nurses, mean (SD) | 2.95 (1.16) | 2.69 (0.99) | <0.001 |
| Operative time, min, mean (SD) | 155.38 (86.96) | 165.22 (85.69) | 0.009 |
| **Cancer type, n (%)** | | | <0.001 |
| Colorectal | 553 (62.6) | 276 (22.0) | |
| Uterine | 287 (32.5) | 186 (14.8) | |
| Prostate | 38 (4.3) | 763 (60.8) | |
| Renal | 6 (0.7) | 30 (2.4) | |
| **T category/FIGO stage group, n (%)** | | | <0.001 |
| T1 or FIGO I | 260 (29.4) | 258 (20.6) | |
| T2 or FIGO II | 167 (18.9) | 413 (32.9) | |
| T3–T4 or FIGO III–IV | 457 (51.7) | 584 (46.5) | |
| Length of hospital stay, days, mean (SD) | 11.12 (7.66) | 7.77 (3.98) | <0.001 |
| 30-day rehospitalization, n (%) | 198 (22.4) | 137 (10.9) | <0.001 |
| 30-day mortality, n (%) | 54 (6.1) | 24 (1.9) | <0.001 |
| Postoperative complications, n (%) | 32 (3.6) | 22 (1.8) | 0.010 |
| Surgical site infection, n (%) | 14 (1.6) | 3 (0.2) | 0.001 |
| Blood transfusion, n (%) | 25 (2.8) | 9 (0.7) | <0.001 |

Continuous variables are presented as mean (SD) and were compared with the Student t test; categorical variables are presented as n (%) and were compared with the Pearson chi-square or Fisher exact test. For variables with more than two levels, a single omnibus test was performed. The laparoscopic and open group comprised 772 laparoscopic and 112 open procedures. SD, standard deviation. T category/FIGO stage groups are study-specific (Supplementary Methods S2). Rehospitalization and mortality were assessed within 30 days after surgery. Complications were identified from pathology test results and were analyzed as an exploratory outcome.

**Table S5B.** Primary cohort characteristics by cancer and surgical approach.

| Characteristic | Colorectal robotic (n=276) | Colorectal laparoscopic (n=514) | Uterine robotic (n=186) | Uterine laparoscopic (n=256) |
|---|---|---|---|---|
| Age, years | 62.2 (11.3) | 68.5 (11.9) | 50.7 (10.5) | 54.8 (13.2) |
| Female sex | 97 (35.1) | 214 (41.6) | 186 (100.0) | 256 (100.0) |
| T1 or FIGO I | 52 (18.8) | 69 (13.4) | 145 (78.0) | 176 (68.8) |
| T2 or FIGO II | 66 (23.9) | 71 (13.8) | 37 (19.9) | 68 (26.6) |
| T3–T4 or FIGO III–IV | 158 (57.2) | 374 (72.8) | 4 (2.2) | 12 (4.7) |
| Operative time, min | 255.6 (116.7) | 192.2 (84.5) | 94.0 (27.7) | 78.0 (25.5) |
| Assisting physicians, n | 0.9 (0.7) | 0.8 (0.7) | 0.5 (0.6) | 0.4 (0.6) |
| Nurses, n | 3.1 (1.3) | 3.1 (1.3) | 2.5 (0.7) | 2.5 (0.7) |
| Length of stay, days | 10.4 (4.7) | 12.9 (7.8) | 4.5 (2.3) | 6.8 (4.1) |

Values are mean (SD) or n (%); denominators are the indicated cancer-by-approach groups. T category/FIGO stage groups are study-specific and are not equivalent TNM/FIGO stages across cancers.

**Table S6.** Cancer-specific adjusted associations, robotic versus laparoscopic surgery.

| Outcome | Colorectal (n = 790) | Uterine (n = 442) |
|---|---|---|
| Length of stay | 0.86 (0.79–0.93) | 0.67 (0.60–0.75) |
| 30-day rehospitalization | 2.50 (1.77–3.53) | 0.38 (0.16–0.90) |
| 30-day mortality | 0.89 (0.44–1.81) | 0.51 (0.05–4.79) |
| Postoperative complications | 0.84 (0.40–1.77) | 0.33 (0.04–2.98) |
| Surgical site infection | 0.35 (0.09–1.29) | Not estimable |

Values are LOS ratios or adjusted odds ratios (95% CI), adjusted for age, sex where variable, and T category/FIGO stage group. Interaction p values were 0.002 for LOS and <0.001 for rehospitalization. Infection estimates are not reported when no events occurred in one treatment group. Complications were identified from pathology test results and were analyzed as an exploratory outcome.

**Table S7.** Primary cohort sensitivity models.

| Outcome | Primary without operative time and staffing | With operative time and staffing | Calendar year adjusted |
|---|---|---|---|
| Length of stay | 0.78 (0.73 to 0.83) | 0.75 (0.70 to 0.80) | 0.78 (0.73 to 0.84) |
| 30-day rehospitalization | 1.82 (1.34 to 2.47) | 1.64 (1.19 to 2.25) | 1.83 (1.35 to 2.49) |
| 30-day mortality | 0.81 (0.42 to 1.60) | 0.62 (0.30 to 1.27) | 0.84 (0.42 to 1.66) |
| Postoperative complications | 0.75 (0.37 to 1.49) | 0.58 (0.28 to 1.22) | 0.76 (0.38 to 1.51) |
| Surgical site infection | 0.29 (0.08 to 1.06) | 0.24 (0.06 to 0.92) | 0.31 (0.08 to 1.12) |

All models use the same 1,232 patients. Values are LOS ratios or odds ratios (95% CI). Primary adjustment excludes operative time and staffing. Calendar year is added to the primary covariates, without operative time or staffing. Complications were identified from pathology test results and were analyzed as an exploratory outcome.

**Table S8.** Department counts and random-intercept sensitivity models.

| Population | Departments | Interpretation |
|---|---|---|
| Four cancers | 3 | Urology, general surgery, obstetrics and gynecology |
| Primary combined | 2 | General surgery and obstetrics and gynecology |
| Each primary cancer group | 1 | Department and cancer are inseparable within the subgroup |

| Outcome | Variance without operative time/staffing | Variance with operative time/staffing | Latent logistic ICC |
|---|---|---|---|
| Length of stay | 0 | 0 | Not applicable |
| 30-day rehospitalization | 0 | 0 | 0 / 0 |
| 30-day mortality | 0 | 0 | 0 / 0 |
| Postoperative complications | $2.68 \times 10^{-20}$ | 0 | $8.15 \times 10^{-21}$ / 0 |
| Surgical site infection | 0 | 0 | 0 / 0 |

ICC pairs show models without/with operative time and staffing. All ten random-intercept models for the primary cohort had boundary variance estimates or numerical estimation problems; surgical-approach estimates are not reported. Boundary estimates are not reliable evidence of no clustering. Surgeon-level effects and volume were not analyzed for ethical and policy reasons. Calendar-year adjustment does not control for surgeon volume or learning curves, and the reported confidence intervals do not account for surgeon-level clustering.

**Table S9.** Standardized outcomes in the secondary four-cancer analysis.

| Outcome | Robotic | Non-robotic | Non-robotic minus robotic (95% CI) |
|---|---|---|---|
| Standardized mean length of stay, days | 8.03 | 10.64 | 2.61 (2.04 to 3.11) |
| Standardized 30-day rehospitalization, % | 17.73 | 14.44 | -3.29 (-6.39 to -0.22) |

Predictions were standardized over the full four-cancer cohort using age, sex, cancer type, and T category/FIGO stage group, without operative time or staffing. Differences equal non-robotic minus robotic; rehospitalization differences are percentage points. Both means and their difference were calculated over the same 2,139 patients. Percentile CIs use 1,000 valid patient-level bootstrap resamples, without surgeon-level clustering. Limited overlap requires extrapolation, so these secondary estimates are not causal effects.
